# Anti-obesity effect of *Solidago virgaurea* var. *g* *igantea* extract through regulation of adipogenesis and lipogenesis pathways in high-fat diet-induced obese mice (C57BL/6N)

**DOI:** 10.1080/16546628.2016.1273479

**Published:** 2017-02-13

**Authors:** Zhiqiang Wang, Ju Hee Kim, Young Soo Jang, Chea Ha Kim, Jae-Yong Lee, Soon Sung Lim

**Affiliations:** ^a^ Department of Food Science and Nutrition, Hallym University, Chuncheon, Republic of Korea; ^b^ Institute of Natural Medicine, Hallym University, Chuncheon, Republic of Korea; ^c^ Department of Biochemistry, Institute of Cell Differentiation and Aging, Hallym University, Chuncheon, Republic of Korea; ^d^ Institute of Korean Nutrition, Hallym University, Republic of Korea

**Keywords:** *Solidago virgaurea* subsp. *gigantea*, anti-obesity, adipogenesis, lipogenesis, lipid profile

## Abstract

**Background:** Obesity is associated with an increase in adipogenesis and is becoming a serious health problem in modern society.

**Objective:** The effects of various Solidago virgaurea var. gigantean (SV) ethanolic aqueous extracts on anti-adipogenesis in 3T3-L1 cells were investigated. In addition, the effect of SV 10% ethanolic extract (SV10E) on preventing obesity was studied in high-fat diet-induced obese mice (C57BL/6 N).

**Design:** The effect of SV10E on preventing obesity was studied in mice (n = 6): normal-fat diet, high-fat diet (HFD), HFD supplemented with 1% (10 g/kg) Garcinia cambogia extract of 60% (–)-hydroxycitric acid (positive control), HFD supplemented with 0.5% (5 g/kg) SV10E, and HFD supplemented with 2% (20 g/kg) SV10E.

**Results:** SV10E showed the highest anti-adipogenic activity in vitro and reduced body weight gain, adipose tissue size, and liver weight, without affecting food intake in vivo. SV10E administration decreased the levels of total triacylglycerol and cholesterol in serum, and lipid metabolites in liver. Adipogenic and lipogenic genes such as PPAR-γ, C/EBP-α, aP2, FAS, SCD-1, SREBP-1c, and CD36 in white adipose tissue and liver were suppressed by SV10E administration.

**Conclusion:** SV10E can be a potent functional food ingredient for preventing HFD-induced obesity by suppressing adipogenesis and lipogenesis.

## Introduction

According to the trends observed in the distribution of body mass index (BMI) among US adults in 1999–2010 by the National Health and Nutrition Examination Survey (NHANES), 75.1% of adults had a BMI of 25 kg/m^2^ or higher, and were considered overweight or obese [1]. Obesity increases the risk of heart disease, high blood pressure, diabetes, arthritis-related disability, and cancer [2]. It is associated with the expansion of adipose tissue, which is induced by adipogenesis and the accumulation of triglycerides in adipocytes [3]. Adipogenesis is a differentiation process by which undifferentiated preadipocytes are converted to mature adipocytes [3,4]. During adipocyte differentiation, adipogenic transcription factors, such as sterol regulatory element-binding protein-1c (SREBP-1c), peroxisome proliferator-activated receptor-γ (PPAR-γ), and CCAAT/enhancer binding protein-α (C/EBP-α), are considered key regulators of adipogenesis [4–6]. SREBP-1c stimulates the expression of C/EBP-α, PPAR-γ, and several other lipogenic enzyme products including fatty acid synthase (FAS) and acetyl-coenzyme (CoA) carboxylase (ACC).

Several plants and their bioactive compounds have been shown to have anti-obesity actions [7], indicating that compounds in plant extracts may provide a novel therapeutic approach for preventing or treating obesity and associated metabolic diseases.

The North-East Asiatic *Solidago virgaurea* complex in Korea has been reported to have five taxa (*S. virgaurea* subsp. *a* *siatica*, *S. virgaurea* var. *t* *aquetii*, *S. virgaurea* var. *c* *oreana*, *S. virgaurea* subsp. *l* *eiocarpa*, and *S. virgaurea* subsp. *g* *igantea*) [8]. *Solidago* plants have been shown to have favorable diuretic, choleretic [9], antiseptic [10], antioxidant, antimicrobial [11], anti-inflammatory [12], antibacterial [13], and wound-healing activities [10]. *Solidago* species contain flavonoids (on average, 1.5% in *S. virgaurea*), anthocyanidins [9], phenolic acids [14]. Other constituents include diterpenes (*cis*- and *trans*-clerodane) [13], bisdesmosidic phenol glycosides such as leiocarposide (0.08–0.48% only in *S. virgaurea*), triterpene saponins of the oleanane type (up to 2%) [15], and essential oil (0.4–1.5%) [16].

In our previous study, we examined the anti-adipogenic effects of *S. virgaurea* var. *g* *igantea* (SV) and three components (protocatechuic acid, chlorogenic acid, and kaempferol-3-*O*-D-rutinoside) isolated from the extracts of SV using Oil Red O dye in 3T3-L1 murine adipocytes [17]. In this study, we investigated the anti-obesity effects of SV administration for 10 weeks, on body weight gain, serum and liver lipid profiles, histological changes in adipose tissue, and expression of the genes associated with adipogenesis and lipogenesis in high-fat diet-induced obese mice.

## Materials and methods

### Plant material and preparation of the extract

SV was harvested from Ulleung Island in May, Republic of Korea. The dried SV (1.5 kg) was crushed and extracts were extracted by an ethanolic aqueous extraction technique (0–100%, 15 l) at 70ºC for 7 h. The extracts, namely SV water extract (SV0E), SV 10% ethanol extract (SV10E), SV 30% ethanol extract (SV30E), SV 50% ethanol extract (SV50E), SV 70% ethanol extract (SV70E), and SV 100% ethanol extract (SV100E), were filtered using filter paper (Hyundai Micro No. 20; Bucheon, Korea). The extracts were then concentrated by a reduced pressure evaporator (N-1000; Tokyo Rikakikai, Tokyo, Japan), and finally freeze-dried using PVTFD10R (Ilshinbiobase Co., Yangju, Korea) to obtain solid extract powder.

### High-performance liquid chromatography analysis

The SV extracts were analyzed using an Agilent 1200 high-performance liquid chromatography (HPLC) system (Agilent Technologies, Santa Clara, CA, USA) with an Eclipse XDB-C18 column (150 × 4.6 mm, 5 μm, Agilent). The mobile phase consisted of A (0.1% trifluoroacetic acid in water) and B (methanol), which was programmed as follows: 0–15 min, 5–40% B; 15–30 min, 40–60% B; 30–40 min, 60–100% B, at a flow rate of 0.7 ml/min. The ultraviolet diode array detector was set at 254 nm and the sample injection volume was 10 μl at a column temperature of 30°C.

### 3T3-L1 cell culture and adipocyte differentiation

3T3-L1 fibroblasts were obtained from the American Type Culture Collection (Manassas, VA, USA) and grown to confluency at 37°C under a humidified 5% carbon dioxide (CO_2_) atmosphere in Dulbecco’s modified Eagle’s medium (DMEM; Gibco, Waltham, MA, USA), containing 10% bovine calf serum (GenDEPOT, Katy, TX, USA), and 100 U/ml penicillin–streptomycin (Gibco). Two days after the cells had reached confluency (day 0), preadipocytes of 3T3-L1 were cultured in differentiation medium (DM) containing 10% fetal bovine serum (FBS; Gibco), 10 μg/ml insulin (Sigma-Aldrich, St. Louis, MO, USA), 0.5 mM 3-isobutyl-1-methyxanthine (IBMX; Sigma-Aldrich), and 1 μM dexamethasone (Sigma-Aldrich). Two days after stimulation with differentiation inducer (MDI; including 0.5 mM IBMX, 1 μM dexamethasone, and 10 μg/ml insulin) (day 2), the medium was switched to post-DM containing 10% FBS and 10 μg/ml insulin. Two days later (day 4), the medium was changed to 10% FBS/DMEM. The cells were cultured in 10% FBS/DMEM every 2 days. Full differentiation was achieved by day 8. The SV extracts were added to the 3T3-L1 culture at the concentration of 10 μg/ml on day 4 after induction of differentiation.

### Oil Red O staining

To determine both adipogenic potential and fat accumulation, the cells were stained with Oil Red O solution (Sigma Chemical Co., St. Louis, MO, USA). On day 8, the cultured 3T3-L1 cells were washed with phosphate-buffered saline (PBS) and then fixed with 10% formaldehyde at room temperature. The cells were stained with 0.5 μg/ml Oil Red O solution. After the Oil Red O staining, cells were photographed using an optical microscope system (Axiomager, Zeiss, Oberkochen, Germany) at 100 × magnification. The lipid droplets were dissolved in isopropanol and absorbance was measured at 540 nm using a microplate reader (Sensident scan; Labsystems, Helsinki, Finland). The relative lipid content and adipogensis inhibitory percentage were calculated using the following equations:

Relative lipid content (%) = (Sample OD/Control OD) × 100%

Inhibition (%) = {1 – (Sample OD – Control OD)/(DM OD – Control OD)} × 100%

where control OD is the absorbance of lipid droplets of the cell population treated without MDI, DM OD is the absorbance of lipid droplets of the cell population treated with MDI, and sample OD is the absorbance of lipid droplets of the cell population treated with MDI and samples.

### MTS assay

The cytotoxicity of SV extracts on 3T3-L1 cells was examined using the 3-(4,5-dimethylthiazol-2-yl)-5-(3-carboxymethoxyphenyl)-2-(4-sulfophenyl)-2H-tetrazolium, inner salt (MTS) assay kit (Promega, Madison, WI, USA). Cells (5 × 10^3^/well) were cultured in 96-well plates and treated with SV extracts (10 μg/ml) for 24 h. After incubation, 20 μl/well of MTS solution was incubated for 20 min at 37°C in a humidified 5% CO_2_ atmosphere. The optical density at 490 nm was measured three times using a microplate reader (Sensident scan).

### Animals and diets

**Figure 1.**
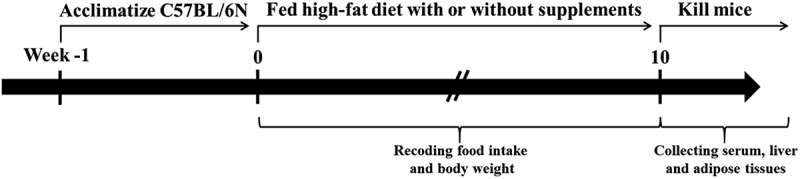
Graphical map of the design of the animal experiment.

All animal experiment procedures were conducted in accordance with the guidelines and approval of the Institutional Animal Care and Use Committees (IACUC) of Hallym University (Hallym R 2015-12). The male C57BL/6 N mice (5 weeks old) were purchased from Central Lab Animal (SLC, Osaka, Japan). All animals were housed in a standardized laboratory environment with a 12 h light/12 h dark cycle at constant room temperature (22 ± 1**°**C) and humidity (60 ± 5%) for 1 week before the experiments for acclimatization. Before being given special diets, the mice were fed standard rodent chow and drinking water ad libitum. After a 1 week acclimation period, according to the rationales of significance in statistics and ethical issues of experimental animals, mice were randomly assigned to five diet groups with six mice in each group (*n* = 6): normal-fat diet (NFD), high-fat diet (HFD), HFD supplemented with 0.5% (5 g/kg) SV10E (SV0.5), HFD supplemented with 2% (20 g/kg) SV10E (SV2), and HFD supplemented with 1% (10 g/kg) *Garcinia cambogia* extract of 60% (–)-hydroxycitric acid (GR; ESFood, Gunpo, Korea). GR was used as a positive control because of its excellent anti-adipogenic and anti-lipogenic properties [18–20]. The experimental diets were based on the AIN-93 diet, and the HFD contained 60% fat (lard 310 g/kg, soybean oil 30 g/kg) to induce obesity. The experimental diets with extract supplements were made by DooYeol Biotech (Seoul, Korea) by mixing diets and corn oil, after dissolving extract supplements in corn oil. The compositions of the diets are shown in Table 1. Fresh diets and water were given every day, and all mice were allowed free access to eat the experimental diets and drink water. During the 10 week experimental period, as shown in Figure 1, body weight was measured once per week and food intake was recorded every day.

**Table 1.** Composition of the experimental diets (g/kg).

Component (g/kg)	Group				
	NFD	HFD	GR	SV0.5	SV2
	
Casein	210	265	265	265	265
L-cystine	3	4	4	4	4
Corn starch	280	–	–	–	–
Maltodextrin	50	160	150	155	140
Sucrose	325	90	90	90	90
Lard	20	310	310	310	310
Soybean oil	20	30	30	30	30
Cellulose	37.15	65.5	65.5	65.5	65.5
Mineral mixture^1^	35	48	48	48	48
Vitamin mixture^2^	15	21	21	21	21
Calcium phosphate, dibasic	2	3.4	3.4	3.4	3.4
Choline bitartrate	2.75	3	3	3	3
Yellow food color	0.1	–	–	–	–
Blue food color	–	0.1	0.1	0.1	0.1
*Garcinia cambogia* extract of 60% (–)-hydroxycitric acid	–	–	10	–	–
*Solidago virgaurea* var. *gigantea* 10% ethanol extract	–	–	–	5	20
Total (g)	1000	1000	1000	1000	1000
Energy (kcal/g)	3.7	5.1	5.1	5.1	5.1
Protein (% of kcal)	20.1	18.3	18.3	18.3	18.3
Carbohydrate (% of kcal)	69.8	21.4	21.4	21.4	21.4
Fat (% of kcal)	10.2	60.3	60.3	60.3	60.3

NFD, normal-fat diet control; HFD, high-fat diet control; GR, HFD + 1% *Garcinia cambogia* extract of 60% (–)-hydroxycitric acid; SV0.5, HFD + 0.5% SV10E; SV2, HFD + 2% SV10E. SV10E, *Solidago virgaurea* var. *g* *igantea* 10% ethanol extract.

^1^ According to AIN-93G-MX (94046).

^2^ According to AIN-93-VX (94047).

### Collection of serum, liver tissue, and adipose tissue

At the end of 10 weeks, all mice were fasted for 12 h, then deeply anesthetized with diethyl ether and killed by cervical dislocation. Blood was collected from the inferior vena cava and separated immediately by centrifugation at 3000 rpm at 4°C for 15 min to isolate the serum. The epididymal adipose tissue and liver were removed, weighed, and stored at 80°C until analysis.

### Biochemical analysis

The serum levels of triacylglycerol (TG), high-density lipoprotein (HDL) cholesterol, total cholesterol (TC), serum alanine aminotransferase (ALT), aspartate aminotransferase (AST), blood urea nitrogen (BUN), and creatinine (CREA) were analyzed using commercial kits (981786, 981823, 981656, 981769, 981771, 981820, and 981811, respectively; Thermo Electron Corporation, Vantaa, Finland) and a Thermo Fisher Konelab 20XTi Analyzer (Thermo Electron Corporation; SeoKwang LABOTECH, Seoul, Korea).

### Histological analysis

Formalin-fixed and paraffin-embedded epididymal adipose tissues were routinely processed for hematoxylin and eosin (H&E) staining. Sections (5 μm thick) were cut and each section was stained with H&E. All sections were examined under an optical microscope (Leica RM2235; Wetzlar, Germany) and printed at a final magnification of 200 ×. Images were visualized using a microscope (Axiomager; Zeiss, Germany) and the area of each adipocyte was determined using AxioVision Rel. 4.8 software (Zeiss).

### RNA extraction, cDNA synthesis, and real-time polymerase chain reaction

Total RNA was isolated from the epididymal adipose tissue using an Easy-Blue kit (Intron Biotechnology, Seoul, Korea) according to the manufacturer’s instructions. Then, total RNA qualification was performed with a NanoDrop-2000 (Thermo Fisher Scientific; Wilmington, DE, USA). Complementary DNA (cDNA) was synthesized (an equal amount of total RNA) with the Moloney murine leukemia virus transcriptase and Oligo (dT) 15 primers (Promega, Madison, WI, USA) using a Life Touch thermal cycler (Life Eco; Bioer Technology, Hangzhou, China). The program was set for 1 h of initiation at 42°C, followed by 10 min of incubation at 95°C and 10 min at 4°C. Real-time polymerase chain reaction (RT-PCR) was performed using the QuantiTect SYBR Green PCR kit (Qiagen, Hilden, Germany), according to the protocol provided by the manufacturer. The cDNA was amplified for 40 cycles of denaturation (95°C for 30 s), annealing (57°C for 40 s), and extension (72°C for 40 s) using a RotorGene RG3000 RT-PCR machine (Corbett Research, Sydney, Australia). The purity of the PCR products was determined on the basis of melting curve analysis. The relative amount of each gene was calculated using the comparative threshold cycle (Ct) method (Applied Biosystems, Waltham, MA, USA). The messenger RNA (mRNA) levels were normalized to β-actin. Primer sequences are shown in Table 2.

**Table 2.** Sequence of primers used in real-time polymerase chain reaction.

Gene	Primer sequence (5′→3′)	
	Forward primer	Reverse primer
β-Actin	GTCGTACCACTGGCATTGTG	GCCATCTCCTGCTCAAAGTC
C/EBP-α	AGACATCAAGCGCCTACATCG	TGTAGGTGCATGGTGGTCTG
PPAR-γ	CCCTGGCAAACGATTTGTAT	AATCCTTGGCCCTCTGAGAT
SREBP-1c	GCGCTACCGGTCTTCTATCA	TGCTGCCAAAAGACAAGGG
CD36	TCCTCTGACATTTGCAGGTCTATC	GTGAATCCAGTTATGGGTTCCAC
SCD-1	CGAGGGTTGGTTGTTGATCTGT	ATAGCACTGTTGGCCCTGGA
FAS	GATCCTGGAACGAGAACAC	AGACTGTGGAACACGGTGGT
aP2	AACACCGAGATTTCCTTCAA	TCACGCCTTTCATAACACAT

### Nuclear magnetic resonance-based hepatic metabolomics

The nuclear magnetic resonance (NMR)-based hepatic metabolomics, including the liver tissue preparation, pulse acquisition, and metabolite identification, and data processing were performed according to previous reports, with minor modification [21]. The lipophilic extracts, which contained most of the lipid constituents of the liver, were used for ^1^H-NMR spectroscopy. The liver tissue (100 mg) was first homogenized in 1 ml chloroform/methanol (CHCl_3_/MeOH, 3:1, v/v). Then, after centrifugation at 10,000 rpm for 10 min at 4°C, the supernatant was collected and dried under a stream of nitrogen. The lipid extract was reconstituted with 665 μl of deuterated chloroform/methanol (CDCl_3_/CD_3_OD, 3:1, v/v) containing tetramethylsilane, which was used as an internal standard in NMR analysis. ^1^H-NMR spectra of lipid extracts were recorded with a Bruker AV 400 instrument (Bruker, Billerica, MA, USA).

### Statistical analysis

Data were expressed as the mean value ± SE and comparisons of data were carried out using the Student’s unpaired *t *test or one-way analysis of variance, as appropriate. *p* < 0.05 was considered statistically significant.

## Results

### Composition of SV extracts

**Figure 2.**
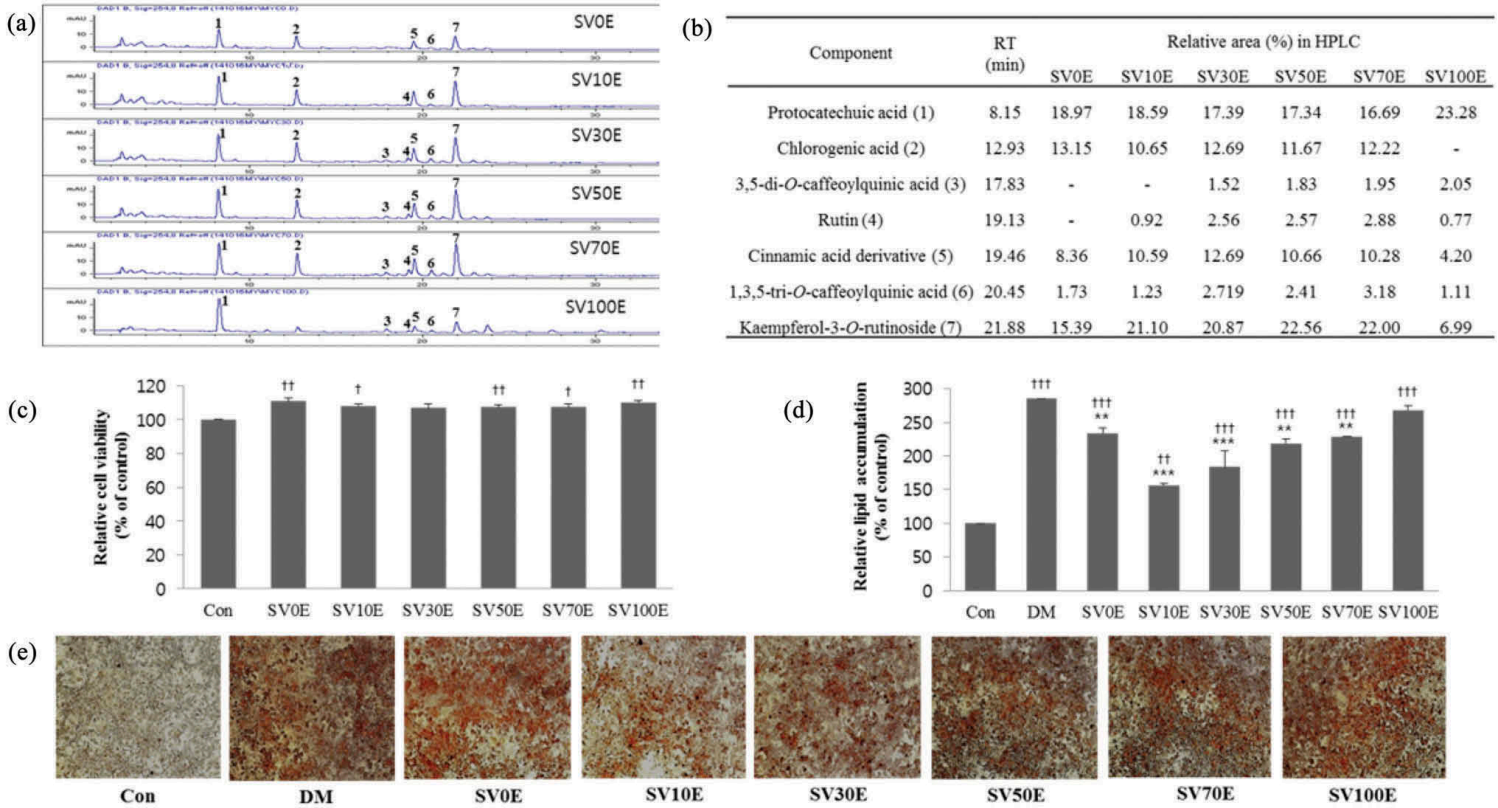
High-performance liquid chromatography (HPLC) profiles of major component of *Solidago virgaurea* var. *gigantea* (SV) ethanolic aqueous extracts and inhibitory effect of SV ethanolic aqueous extracts on 3T3-L1 adipocyte differentiation. (a) Chromatograms of SV ethanolic aqueous extracts; (b) contents of major components of SV ethanolic aqueous extracts; (c) effect of SV ethanolic aqueous extracts on viabilities of 3T3-L1 cells as determined by 3-(4,5-dimethylthiazol-2-yl)-5-(3-carboxymethoxyphenyl)-2-(4-sulfophenyl)-2H-tetrazolium (MTS) assay at 10 μg/ml for 24 h; (d) relative lipid content quantified by absorbance of 3T3-L1 cells treated with or without SV ethanolic aqueous extract at 10 μg/ml for 8 days; (e) microscope images of lipid droplets stained with Oil Red O in 3T3-L1 cells treated with or without SV ethanolic aqueous extract at 10 μg/ml for 8 days. Results are presented as mean ± SE. Asterisks indicate significant differences from differentiation medium (DM) (*p < 0.05, **p < 0.01, ***p < 0.001); daggers indicate significant differences from control (Con) (†*p* < 0.05, ††*p* < 0.01, †††*p* < 0.001). 1, protocatechuic acid; 2, chlorogenic acid; 3, 3,5-di-*O*-caffeoylquinic acid; 4, rutin; 5, unknown cinnamic acid derivative compound; 6, 1,3,5-tri-*O*-caffeoylquinic acid; 7, kaempferol-3-*O*-rutinoside; RT, retention time; SV0E, SV water extract; SV10E, SV 10% ethanol extract; SV30E, SV 30% ethanol extract; SV50E, SV 50% ethanol extract; SV70E, SV 70% ethanol extract; SV100E, SV 100% ethanol extract.

The SV was extracted with 0%, 5%, 10%, 30%, 50%, 70%, and 100% aqueous ethanol. Their compositions were compared by HPLC. As shown in Figure 2(a,b), their compositions were similar, but the content (amount) of each component was different. SV0E had the highest content of chlorogenic acid, SV30E had the highest content of one unknown compound, SV50E had the highest content of kaempferol-3-*O*-rutinoside, SV70E had the highest content of rutin and 1,3,5-tri-*O*-caffeoylquinic acid, and SV100E had the highest content of protocatechuic acid and 3,5-di-*O*-caffeoylquinic acid.

### Effect of SV extracts on preadipocyte viability

An MTS assay was performed to assess the effects of different SV extracts on 3T3-L1 cell viability. As shown in Figure 2(c), the SV extracts at 10 μg/ml had no significant effects on viability after 24 h treatment.

### Inhibitory effects of various SV extracts on lipid accumulation in 3T3-L1 cells

The anti-adipogenic effects of different SV extracts were compared using Oil Red O staining in 3T3-L1 cells at a concentration of 10 μg/ml. As shown in Figure 2(d,e), lipid contents in 3T3-L1 adipocytes decreased significantly following treatment with SV0E, SV10E, SV50E, and SV70E. Among them, SV10E exhibited the highest inhibitory effect (57.69%) on adipogenesis.

### Changes in body weight, food intake, and food efficiency ratio

**Figure 3.**
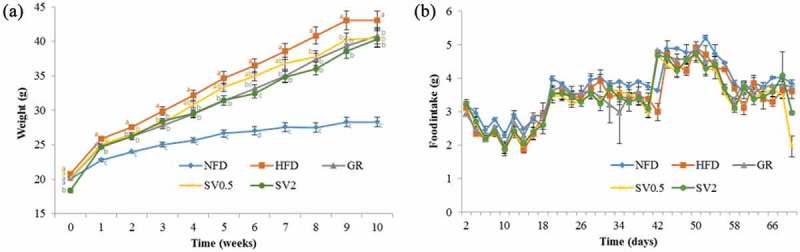
Effects of *Solidago virgaurea* var. *gigantea* 10% ethanol extract (SV10E) on (a) body weight and (b) food intake in high-fat diet-induced obese mice. Results are presented mean ± SE (*n* = 6). NFD, normal-fat diet control; HFD, high-fat diet control; GR, HFD + 1% *Garcinia cambogia* extract of 60% (–)-hydroxycitric acid; SV0.5, HFD + 0.5% SV10E; SV2, HFD + 2% SV10E. Points in the same week with different letters are significantly different from each other (*p* < 0.05).

SV10E exhibited the maximum anti-adipogenic effect on 3T3-L1 cells without affecting cell viability; therefore, it was used for further study on preventing obesity *in vivo*. The study design is shown in Figure 1, and the compositions of the experimental diets are listed in Table 1. As shown in Figure 3(b), the differences in food intake between each group were not significant during the 10 week feeding period. For body weights (Figure 3(a)), there were no significant difference initially; after 10 weeks of experimental diet feeding, the body weights of HFD mice increased to 1.52 times those of NFD mice; however, SV10E supplementation for 10 weeks significantly decreased the body weights in comparison to the HFD group. As shown in Table 3, the body weight gain and food efficiency ratio [FER = Body weight gain (g/day)/Food intake (g/day)] were suppressed by SV10E, but SV10E had minimal effect on food intake.

**Table 3.** Effects of *Solidago virgaurea* var. *gigantea* 10% ethanol extract (SV10E) on body weight in mice fed a high-fat diet.

	Group				
	NFD	HFD	GR	SV0.5	SV2
Body weight gain (g/10 weeks)	8.29 ± 0.74^a^	24.05 ± 1.28^c^	19.27 ± 1.28^b^	20.28 ± 1.35^b^	21.54 ± 1.68^b^
Food intake (g/day)	3.74 ± 0.13^a^	3.41 ± 0.13^a^	3.51 ± 0.13^a^	3.33 ± 0.13^a^	3.40 ± 0.13^a^
FER	2.22 ± 0.20^a^	7.06 ± 5.49^ab^	5.49 ± 0.36^b^	6.09 ± 0.41^b^	6.34 ± 0.50^b^
Epididymal white adipose tissue weight (g)	1.00 ± 0.16^a^	2.31 ± 0.16^b^	2.18 ± 0.16^b^	2.53 ± 0.28^b^	2.36 ± 0.08^b^
Liver weight (g)	1.27 ± 0.19^a^	1.65 ± 0.38^b^	1.19 ± 0.07^a^	1.34 ± 0.09^a^	1.18 ± 0.04^a^

Results are presented as mean ± SE (*n* = 6). Values within a row with different letters are significantly different from each other (*p* < 0.05).

NFD, normal-fat diet control; HFD, high-fat diet control; GR, HFD + 1% *Garcinia cambogia* extract of 60% (–)-hydroxycitric acid; SV0.5, HFD + 0.5% SV10E; SV2, HFD + 2% SV10E; FER, food efficiency ratio = Body weight gain (g/day)/Food intake (g/day).

### Serum lipid profiles

The effects of HFD and SV10E administration on serum lipid profiles are shown in Table 4. The serum TG and TC levels increased in the HFD group in comparison to those in NFD group, but a high concentration of SV10E significantly reduced the serum TG and TC levels by 33.91% and 45.79%, respectively, compared to those of the HFD group. Although SV10E decreased the HDL-cholesterol level in comparison to the HFD group, the HDL-cholesterol/Total cholesterol ratio (HTR) was not influenced significantly. The toxicity of SV10E administration was determined by measuring serum AST, ALT, BUN, and CREA levels. Serum AST, ALT, BUN, and CREA levels increased following HFD feeding, whereas high-concentration SV10E administration decreased AST, ALT, and CREA levels compared to the HFD group. Serum BUN did not increase during the feeding period, whereas a high concentration of SV reduced 53% of CREA in comparison to the HFD group.

**Table 4.** Effects of *Solidago virgaurea* var. *gigantea* 10% ethanol extract (SV10E) on obese biomarkers in mice fed a high-fat diet.

	Group				
	NFD	HFD	GR	SV0.5	SV2
Serum TG (mg/dl)	115.50 ± 11.60^b^	120.90 ± 13.70^b^	120.30 ± 8.40^b^	114.20 ± 8.70^b^	79.90 ± 8.00^a^
Serum total cholesterol (mg/dl)	53.52 ± 4.23^a^	118.10 ± 5.67^b^	127.12 ± 8.30^b^	133.51 ± 6.47^b^	64.02 ± 3.33^a^
Serum HDL-cholesterol (mg/dl)	43.17 ± 2.79^a^	78.81 ± 3.16^b^	79.45 ± 2.38^b^	83.44 ± 3.37^b^	42.51 ± 1.65^a^
HTR	0.81 ± 0.02^c^	0.70 ± 0.01^b^	0.66 ± 0.02^b^	0.67 ± 0.01^b^	0.66 ± 0.01^b^
AST (U/l)	66.54 ± 15.29^ab^	79.61 ± 15.78^ab^	113.69 ± 15.75^bc^	135.98 ± 16.67^c^	43.63 ± 5.15^a^
ALT (U/l)	31.85 ± 7.75^ab^	89.52 ± 22.90^bcd^	70.43 ± 11.76^c^	132.72 ± 22.03^d^	21.86 ± 1.75^a^
BUN (mg/dl)	22.55 ± 0.78^c^	17.49 ± 0.32^b^	17.73 ± 0.47^b^	19.30 ± 0.86^b^	6.76 ± 0.72^a^
CREA (mg/dl)	0.41 ± 0.02^bc^	0.45 ± 0.01^c^	0.42 ± 0.01^bc^	0.41 ± 0.01^b^	0.21 ± 0.00^a^

Results are presented as mean ± SE (*n* = 6). Values within a row with different letters are significantly different from each other (*p* < 0.05).

NFD, normal-fat diet control; HFD, high-fat diet control; GR, HFD + 1% *Garcinia cambogia* extract of 60% (–)-hydroxycitric acid; SV0.5, HFD + 0.5% SV10E; SV2, HFD + 2% SV10E; TG, triacylglycerol; HDL, high-density lipoprotein; HTR, HDL-cholesterol/Total cholesterol ratio; AST, aspartate aminotransferase; ALT, alanine aminotransferase; BUN, blood urea nitrogen; CREA, creatinine.

### Changes in weight and morphology of adipose tissue

**Figure 4.**
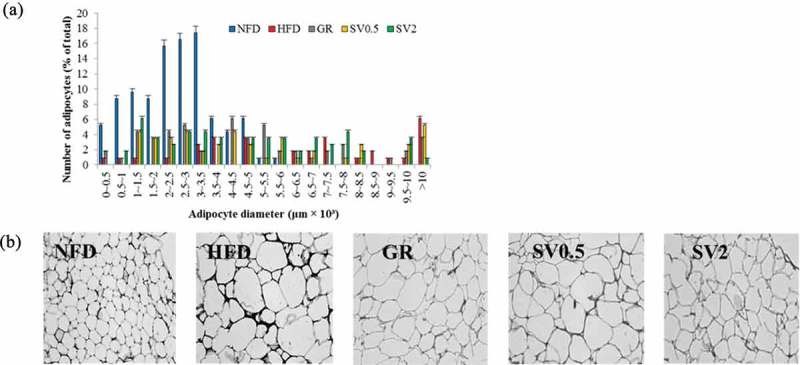
Effects of *Solidago virgaurea* var. *gigantea* 10% ethanol extract (SV10E) on (a) epididymal white adipose tissue diameter and (b) morphology in high-fat diet-induced obese mice. Results are presented as mean ± SE (*n* = 3). Each specimen from the mouse groups was fixed with 4% paraformaldehyde and sectioned at 5 μm, stained with hematoxylin and eosin (H&E), and then viewed using light microscopy at a magnification of 200 ×. The image is a representative one. NFD, normal-fat diet control; HFD, high-fat diet control; GR, HFD + 1% *Garcinia cambogia* extract of 60% (–)-hydroxycitric acid; SV0.5, HFD + 0.5% SV10E; SV2, HFD + 2% SV10E.

To determine whether the weight-reducing effect of SV10E was due to the decrease in fat mass, the adipocyte size and tissue weight were measured. The weight of epididymal white adipose tissue in the HFD group increased significantly in comparison to that of the NFD group (Table 3), and the adipocyte size was larger in the HFD group (the major adipocyte diameter was over 10,000 μm) in comparison to that of the NFD group (the major adipocyte diameter was below 3500 μm) (Figure 4). Supplementation with a high concentration of SV10E decreased adipocyte size clearly, although it did not influence the white adipose tissue weight (Table 3 and Figure 4).

### Effect of SV10E on expression of genes related to adipogenesis in white adipose tissue

**Figure 5.**
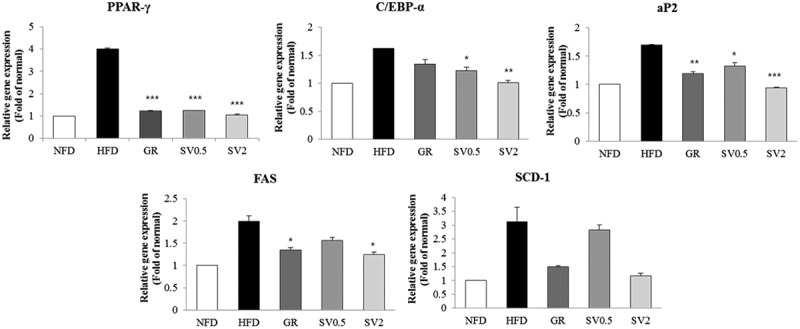
Effects of *Solidago virgaurea* var. *gigantea* 10% ethanol extract (SV10E) on mRNA expression of adipogenic genes in epididymal white adipose tissue. Results are presented as mean ± SE (*n* = 3). Asterisks indicate significant differences from the HFD group (*p < 0.05, **p < 0.01, ***p < 0.001). NFD, normal-fat diet control; HFD, high-fat diet control; GR, HFD + 1% *Garcinia cambogia* extract of 60% (–)-hydroxycitric acid; SV0.5, HFD + 0.5% SV10E; SV2, HFD + 2% SV10E.

To further evaluate SV10E-induced reduction in adipocyte size, we measured the mRNA expression of markers related to adipogenesis and lipogenesis. As shown in Figure 5, supplementation with a high concentration of SV10E significantly reduced the expression of adipogenic mRNAs such as PPAR-γ, C/EBP-α, and fatty acid binding protein-4 (aP2). The expression of lipogenic mRNAs, FAS, and stearoyl-CoA desaturase-1 (SCD-1) was also down-regulated by SV10E.

### Changes in liver weight and hepatic lipid metabolites

Because obesity is often accompanied by the development of fatty liver, we examined the effect of SV10E on liver weight changes and hepatic lipid metabolites in HFD-fed mice. The liver weight was higher in the HFD group than in the NFD group, and SV10E supplementation decreased liver weight significantly (Table 3). Four types of hepatic lipid metabolites, namely fatty acids, phospholipid, lipid moieties, and cholesterol, were analyzed, and their levels increased significantly in the HFD group (Table 5). Supplementation with a high concentration of SV10E significantly decreased the level of hepatic lipid metabolites. These collective results suggested that SV10E effectively lowered lipid accumulation in the liver.

**Table 5.** ^1^H-Nuclear magnetic resonance chemical shifts for endogenous lipid-soluble hepatic metabolites.


	δ^1^H (ppm)	Type	Metabolites		Group				
					NFD	HFD	GR	SV0.5	SV2
(A)	5.32	Fatty acids	MUFA/PUFA		7.78 ± 0.80^a^	29.8 ± 4.38^b^	9.63 ± 1.76^ab^	7.07 ± 0.17^a^	5.32 ± 0.65^a^
(B)	3.16	Phospholipids	N^+^(CH_3_)_3_		0.18 ± 0.00^a^	2.11 ± 0.34^c^	0.33 ± 0.00^b^	0.28 ± 0.03^ab^	0.19 ± 0.01^a^
(C)	2.78	Lipid moieties	–CH=CH(–CH_2_CH=CH–)	}	3.56 ± 0.29^a^	18.2 ± 4.98^b^	3.96 ± 0.14^a^	3.51 ± 0.05^a^	2.70 ± 0.41^a^
(D)	2.72		–CH=CH–CH_2_CH=CH–						
(E)	2.27		αRCH_2_CH_2_CO		7.40 ± 1.02^a^	31.67 ± 9.92^b^	10.61 ± 2.46^a^	7.99 ± 0.96^a^	4.80 ± 0.49^a^
(F)	2		–CH_2_CH=CH–		6.92 ± 0.43^a^	39.22 ± 11.61^b^	12.91 ± 3.26^a^	10.16 ± 1.10^a^	6.54 ± 0.59^a^
(G)	1.55		βRCH_2_CH_2_CO		6.49 ± 0.20^a^	23.61 ± 2.53^b^	10.17 ± 2.48^a^	7.71 ± 0.79^a^	4.38 ± 0.68^a^
(H)	1.24		–(CH_2_)*_n_*–		54.65 ± 3.47^a^	201.94 ± 32.30^b^	78.24 ± 21.31^ab^	60.92 ± 8.48^a^	36.05 ± 5.40^a^
(I)	0.83		RCH_3_		4.60 ± 0.55^a^	33.57 ± 7.76^b^	10.63 ± 2.27^a^	8.42 ± 0.89^a^	5.50 ± 0.85^a^
(J)	0.64	Cholesterol	Chol-C18		0.05 ± 0.03^a^	0.51 ± 0.35^c^	0.20 ± 0.73^b^	0.18 ± 0.03^b^	0.04 ± 0.03^a^

Results are presented as mean ± SE (*n* = 6). Values within a row with different letters are significantly different from each other (*p* < 0.05).

NFD, normal-fat diet control; HFD, high-fat diet control; GR, HFD + 1% *Garcinia cambogia* extract of 60% (–)-hydroxycitric acid; SV0.5, HFD + 0.5% SV10E; SV2, HFD + 2% SV10E. SV10E, *Solidago virgaurea* var. *g* *igantea* 10% ethanol extract; MUFA, monounsaturated fatty acid; PUFA, polyunsaturated fatty acid.

### Effect of SV10E on expression of genes related to lipogenesis in the liver

**Figure 6.**
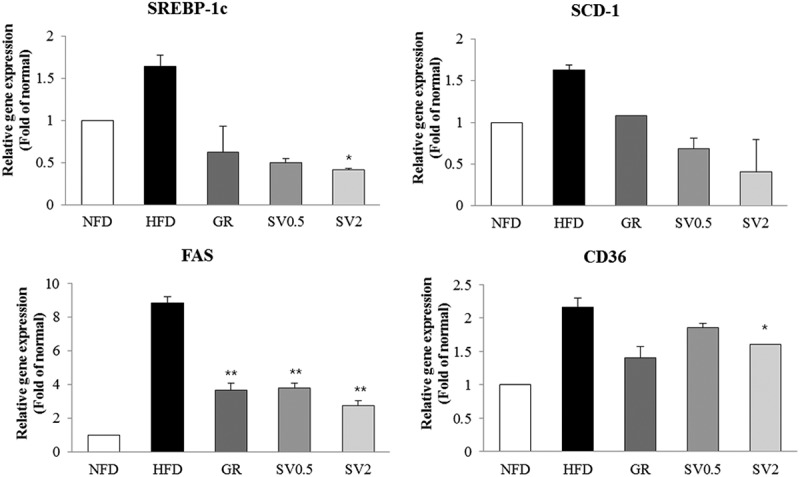
Effects of *Solidago virgaurea* var. *gigantea* 10% ethanol extract (SV10E) extract on mRNA expression of lipogenesis-related genes in the liver. Results are presented as mean ± SE (*n* = 3). Asterisks indicate significant differences from the HFD group (*p < 0.05, **p < 0.01). NFD, normal-fat diet control; HFD, high-fat diet control; GR, HFD + 1% *Garcinia cambogia* extract of 60% (–)-hydroxycitric acid; SV0.5, HFD + 0.5% SV10E; SV2, HFD + 2% SV10E.

To further evaluate the SV10E-induced reduction in hepatic lipid accumulation, we measured the mRNA expression of markers related to lipogenesis. SV10E supplementation significantly reduced the expression of lipogenesis-related mRNAs such as SREBP-1c, FAS, SCD-1, and fatty acid translocase (CD36) (Figure 6).

## Discussion

In modern society, obesity leads to many life-threatening health problems including hypertension, hyperlipidemia, cardiovascular diseases, diabetes, cancers, and non-alcoholic fatty liver diseases [22]. Obesity is associated with the expansion of adipose tissue, which is induced by adipogenesis and the accumulation of triglycerides through lipogenesis as the energy source in adipocytes. Thus, to prevent obesity, dietary supplements are used for regulation of adipogenesis and lipogenesis. Because the currently available drugs for preventing obesity cause serious side effects, a growing demand exists for therapeutically potent but less cytotoxic anti-obesity drugs. In addition, natural products have been used worldwide as traditional herbal medicines to treat different diseases, including obesity and its related metabolic disorders [23]. In a previous study, we found that SV has significant anti-adipogenic and anti-lipogenic effects on 3T3-L1 cells [17]. In that study, one interesting phenomenon was that the fractionation-guided isolated compounds from SV, including keampferol-3-*O*-rutinoside, chlorogenic acid, and protocatechuic acid, showed less anti-adipogenic activity than SV crude extracts. These results seem to indicate a synergistic effect on adipogenesis by the SV components. Thus, SV could be a potential food supplement for preventing obesity, and its anti-obesity effect was investigated in this study.

The extraction conditions can influence bioactive ingredients, resulting in different activities. Therefore, various extracting solvents were applied to evaluate the biological actions of SV on adipogenesis in 3T3-L1 cells, and SV10E showed the highest anti-adipogenic effect at a concentration of 10 μg/ml without cytotoxicity. To determine the difference between SV10E and other extracts, HPLC was performed; however, no difference was observed. Although their activities are completely different, the components in different extracts are similar. Initially, we thought that these high or low activities would be induced by the high concentrations of any active components; however, the content of each component seemed to change in redox. As suggested previously, a synergistic effect on anti-adipogenesis was exerted by the SV extracts, making SV10E a potential anti-adipogenic drug. Therefore, the effect of SV10E on preventing obesity was investigated in HFD-induced obese mice.

The prevalence of obesity has rapidly increased in the past several decades because of changes in lifestyle, and an HFD is considered as the major risk factor [24]. Administration of HFD enhanced body weight gain along with elevation of plasma TG, TC, AST, and ALT, and reduction of HTR. SV10E clearly decreased the body weight gain and FER level in comparison to the HFD group, but it did not affect food intake, indicating that the body weight-lowering effect of SV did not affect appetite. Accordingly, administration of a high concentration of SV10E also significantly decreased the serum TG, TC, AST, and ALT levels. The reduction of weight gain and serum lipids induced by SV10E administration can be correlated with the inhibition of abnormal fat accumulation in adipose tissue and hepatic tissue.

**Figure 7.**
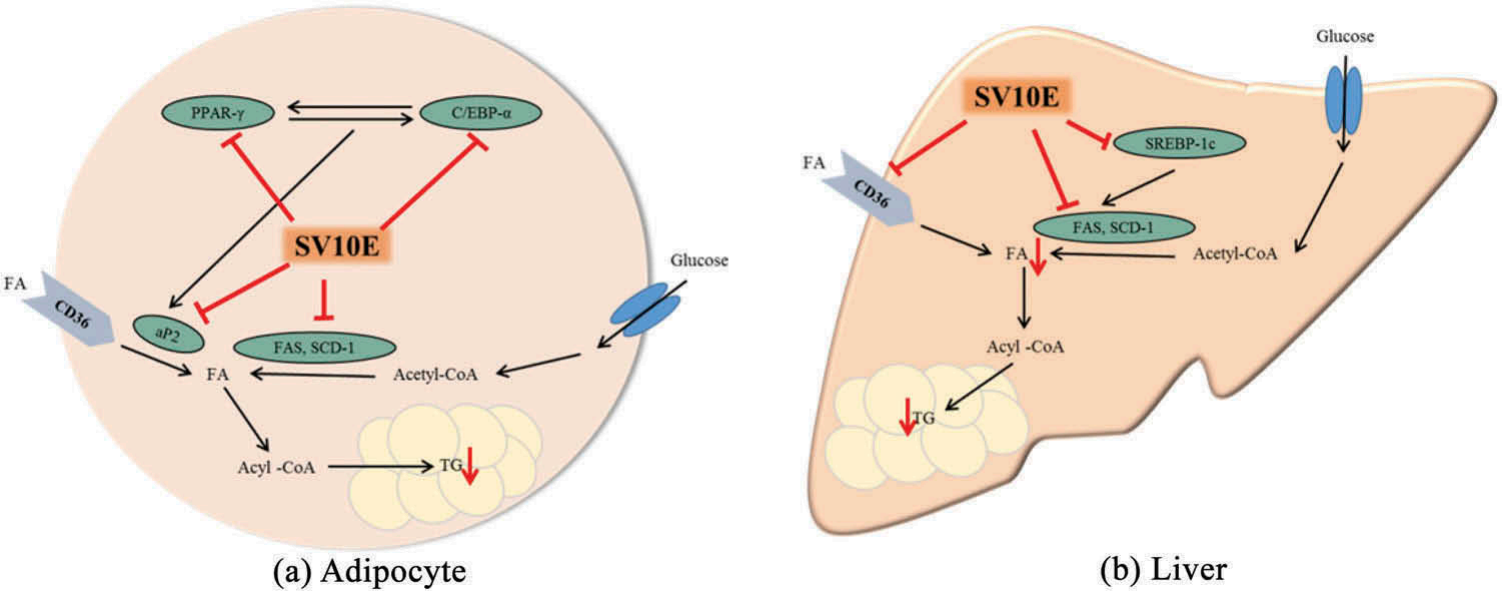
Proposed mechanisms of the anti-obesity effect of *Solidago virgaurea* var. *gigantea* 10% ethanol extract (SV10E) in (a) adipocyte and (b) liver. PPAR-γ, peroxisome proliferator-activated receptor-γ; C/EBP-α, CCAAT/enhancer binding protein-α; aP2, fatty acid binding protein-4; SREBP-1c, sterol regulatory element-binding protein-1c; FAS, fatty acid synthase; SCD-1, stearoyl-CoA desaturase-1; CD36, fatty acid translocase; FA, fatty acid; TG, triacylglycerol.

Obesity is characterized by an increase in fat cell numbers and size, either separately or in combination. Even though SV10E did not affect the epidermal white adipose tissue mass, it significantly decreased the adipocyte size. Adipocytes are the major cellular component of adipose tissue. Many studies have reported that obesity has been prevented by reducing the differentiation of fibroblastic preadipocytes to mature adipocytes (adipogenesis) and inhibiting lipogenesis [25]. Adipocyte differentiation is a multistep process that is regulated by a network of transcription factors and adipogenesis-related genes. Adipose tissue regulates fat cell development by coordinated binding of the transcription factors in the regulatory regions of adipogenesis-related genes. The nuclear hormone receptor PPARs are a representative transcription factor family which has been implicated in that process. During adipogenesis, PPAR-γ is considered to be the major regulator of adipocyte differentiation and, along with C/EBP-α, it regulates the expression of downstream target genes [26]. The HFD stimulates expression of PPAR-γ and C/EBP-α, which operate in a self-regulating positive feedback loop system to increase the expression of genes related to adipogenesis and activate the expression of lipid-metabolizing enzymes such as aP2, FAS, and SCD-1, leading to morphological changes and lipid accumulation in cells [27]. aP2 is a carrier protein for fatty acids that is mainly expressed in adipocytes and macrophages and plays an important role in the development of insulin resistance and atherosclerosis in relation to metaflammation. aP2 is elevated by obesity and is used as a marker for adipocyte differentiation [28]. However, the mRNA expression of aP2 in adipose tissue elevated by HFD was reduced by administration of SV10E, implying that the adipocyte differentiation may be prevented. Moreover, the decreased levels of mRNA expression of PPAR-γ and C/EBP-α after administration of SV10E also confirmed the prevention of adipogenesis. FAS and SCD-1, which are the key enzymes in fatty acid metabolism responsible for synthesizing palmitate (C16:0) from acetyl-CoA and forming a double bond in stearoyl-CoA, were also reduced by administration of SV10E. These results strongly suggest that SV10E reduced adipogenesis and lipogenesis via decreased expression of adipogenic mRNAs (Figure 7(a)).

Hepatic tissue is another important target for the prevention of obesity. Because increased plasma lipids in the liver lead to hepatic lipid accumulation through increased hepatic fatty acid synthesis [29], obesity is often accompanied by the development of fatty liver; the decreased serum lipids induced by SV10E administration decreased serum lipid levels, indicating that SV10E may prevent hepatic lipid accumulation. Mobilization of lipids through lipogenesis causes an increase in fat mass and is accompanied by weight gain [30], whereas the liver weight was clearly decreased by SV10E in the present study. In addition, in the lipogenic process of the liver, fatty acids are esterified with glycerol to form TG, or with cholesterol and cholesteryl esters that accumulate and lead to increased hepatic lipid content or fatty acids, whereas our results showed that the hepatic lipid metabolites were also decreased by SV10E administration. The SREBPs are a representative transcription factor family that has been implicated in that process. SREBP-1c is considered as a transcription factor that regulates the expression of downstream target genes involved in glucose utilization and fatty acid synthesis, such as FAS and SCD-1 [31]. The present study showed that lipogenesis-related mRNAs such as FAS, SCD-1, and SREBP-1c were markedly suppressed following SV10E treatment (Figure 7(b)). FAS and SCD-1 are the central lipogenic proteins that, along with CD36, which is an integral membrane protein importing fatty acids inside cells, contribute to energy storage by increasing fatty acid uptake in the liver. Thus, the lipogenesis-inhibiting effect of SV10E may be mediated by repressing fatty acid uptake via inhibition of SREBP-1c, FAS, SCD-1, and CD36. Taken together, these results indicate that SV10E supplementation helps to prevent hepatic fat accumulation through the modulation of lipogenesis.

## Conclusion

In summary, SV10E showed an obesity-preventing effect along with decreased lipid accumulation in adipose tissue and hepatic tissue in male C57BL/6 N mice. SV10E also decreased expression of adipogenic and lipogenic mRNA in white adipose tissue and hepatic tissue of male mice, respectively. Thus, SV10E exerts obesity-preventing effects by suppressing adipogenesis through decreased expression of adipogenesis-related genes and modulating the expression of lipogenesis-related genes in male mice. Hence, SV10E could be a potent and useful functional food ingredient, and further work with female mice would be warranted to confirm similar effects.
